# The Human Replicative Helicase, the CMG Complex, as a Target for Anti-cancer Therapy

**DOI:** 10.3389/fmolb.2018.00026

**Published:** 2018-03-29

**Authors:** Yeon-Soo Seo, Young-Hoon Kang

**Affiliations:** ^1^Department of Biological Sciences, Korea Advanced Institute of Science and Technology, Daejeon, South Korea; ^2^Core Protein Resources Center, Daegu Gyeongbuk Institute of Science and Technology, Daegu, South Korea

**Keywords:** CMG, DNA helicase, DNA replication, cancer, therapeutic target

## Abstract

DNA helicases unwind or rearrange duplex DNA during replication, recombination and repair. Helicases of many pathogenic organisms such as viruses, bacteria, and protozoa have been studied as potential therapeutic targets to treat infectious diseases, and human DNA helicases as potential targets for anti-cancer therapy. DNA replication machineries perform essential tasks duplicating genome in every cell cycle, and one of the important functions of these machineries are played by DNA helicases. Replicative helicases are usually multi-subunit protein complexes, and the minimal complex active as eukaryotic replicative helicase is composed of 11 subunits, requiring a functional assembly of two subcomplexes and one protein. The hetero-hexameric MCM2-7 helicase is activated by forming a complex with Cdc45 and the hetero-tetrameric GINS complex; the Cdc45-Mcm2-7-GINS (CMG) complex. The CMG complex can be a potential target for a treatment of cancer and the feasibility of this replicative helicase as a therapeutic target has been tested recently. Several different strategies have been implemented and are under active investigations to interfere with helicase activity of the CMG complex. This review focuses on the molecular function of the CMG helicase during DNA replication and its relevance to cancers based on data published in the literature. In addition, current efforts made to identify small molecules inhibiting the CMG helicase to develop anti-cancer therapeutic strategies were summarized, with new perspectives to advance the discovery of the CMG-targeting drugs.

## DNA helicases, an emerging target for cancer therapy

DNA helicases unwind duplex DNA in an ATP hydrolysis-dependent manner. *In silico* studies have identified 31 human DNA helicases that are functionally non-redundant (Umate et al., 2011). They are involved in a variety of DNA transactions including DNA replication, repair, recombination and telomere maintenance for the preservation of genome stability (Brosh, 2013; Bochman, 2014; Croteau et al., 2014). Failure of faithful duplication and repair of DNA leads to the loss of genome integrity, thereby causing cancers or other diseases which promote cancers. Many helicase-linked diseases have been identified (Bochman, 2014). Thus, DNA helicases have important roles as genome caretakers and can be considered as potential targets for treating life-threatening diseases including cancers.

The initial effort to discover potent helicase inhibitors had been put to control the replication of infectious microorganisms. Compounds that inhibit helicases have been explored to find alternative way to treat immunocompromised patients suffering from cold sores and genital herpes caused by herpes simplex virus (HSV) infection (Crute et al., 2002; Kleymann et al., 2002). Antiviral drugs targeting viral DNA polymerase (including guanosine, nucleotides or pyrophosphate analog) have been developed over the past 40 years and widely used. However, discovery of the drug-resistant isolates has required development of novel strategies to treat HSV patients (Jiang et al., 2016). The HSV helicase-primase complex is composed three subunits possessing 5′–3′ helicase, primase, and single stranded DNA (ssDNA)-dependent NTPase activities that are essential for DNA synthesis. Several compounds inhibiting the helicase-primase complex with notable efficacy have been identified, although viral resistance against some of these molecules have been reported (James et al., 2015). Two of them (Pritelivir and Amenamevir) have been applied for human clinical trials. Other viral DNA helicases including papillomavirus E1 and polyomavirus large tumor antigen (Tag) are also widely studied as drug targets (Shadrick et al., 2013).

The non-structural protein 3 (NS3) of Hepatitis C virus (HCV) is another example of DNA helicase as an antiviral drug target. NS3 RNA helicase, which also possesses DNA helicase activity, is responsible for viral genome replication (Pang et al., 2002). Nearly 200 million people are estimated to be infected by HCV worldwide and effective vaccine is not currently available (Jin et al., 2017). Several compounds that inhibit the NS3 helicase activity have been isolated and the most potent compound tested inhibited unwinding activity of NS3 by more than 50% at approximately 2 μM concentration (Jin et al., 2017).

The strategy to develop antibacterial drugs by targeting DNA helicases is also relatively novel concept in comparison to other types of antibiotics. The mechanistic studies of DNA replication with *in vitro* reconstitution systems have been extensively carried out using purified proteins from *Escherichia coli (E. coli)*. Isolation and biochemical analysis of *E. coli* DnaB replicative helicase enabled the reconstitution of *E. coli* replisome *in vitro* and has deepened our understanding on the prokaryotic DNA replication (Wright et al., 1973; LeBowitz and McMacken, 1986; Yao and O'Donnell, 2010). Inhibitors against DnaB could be used to treat pathogenic strains of *E. coli*. Several groups identified inhibitors of DnaB helicase of *Bacillus anthracis (B. anthracis)* and *Staphylococcus aureus (S. aureus)* through the use of high-throughput screenings (Head et al., 2016). The most potent and selective molecule inhibited DnaB helicase activities of *B. anthracis* and *S. aureus* with an IC_50_ of 0.2 μM (Li et al., 2013). *B. anthracis* as a potential biological warfare and methicillin-resistant *S. aureus* (MRSA) infections among patients in the emergency department, have made it urgent to develop novel therapies for the treatment of these bacteria (Shadrick et al., 2013; Head et al., 2016).

Malaria is one of the widespread human diseases cause by the parasite *Plasmodium falciparum (P. falciparum)*. Approximately, 212 million cases occurred worldwide in 2015, leading to 429,000 deaths (WHO, 2016). It was shown that some compounds inhibited activity of a *P. falciparum* helicase and the growth of the parasite (Tuteja, 2007). Genome analyses have revealed that *P. falciparum* contains several parasite-specific helicases in addition to homolog of human helicases. Development of inhibitors that specifically target the helicases unique to *P. falciparum* would help to cure malaria (Tuteja, 2017).

The similar idea has been applied to our own DNA helicases in order to curb uncontrolled growth of cancers. Our combats against cancers have come a long way and numerous kinds of drugs have been developed based on understandings of the mechanisms governing proliferation of normal and cancerous cells and tumor progression. These include a vast array of cellular processes such as cell cycle checkpoint, transcription, microtubule assembly, DNA replication and repair, signal transduction, angiogenesis, ubiquitin proteasome system, and immune checkpoint (Dickson and Schwartz, 2009; Priyadarshini and Keerthi, 2012; Vasudev and Reynolds, 2014; Weathington and Mallampalli, 2014; Gross et al., 2015; Sharma and Allison, 2015; Gavande et al., 2016; Zhang et al., 2016). Developing anti-cancer therapy by targeting DNA repair pathways has been an active area of recent cancer research with a promising future. One of the examples is olaparib, an inhibitor of poly(ADP-ribose) polymerase (PARP) which is involved in multiple DNA repair pathways by recruiting repair proteins (Gavande et al., 2016). Olaparib was approved by FDA in December 2014 as monotherapy for ovarian cancer. Finding potent and safe inhibitors against DNA processing enzymes, DNA binding proteins, DNA polymerases and DNA damage response kinases acting on DNA repair pathways are also under extensive investigations. DNA helicases belong to the category of the DNA processing enzymes.

Several model DNA repair pathways and related DNA helicases as prospective targets for treatment of cancers were discussed comprehensively in several reviews (Gupta and Brosh, 2008; Brosh, 2013; Suhasini and Brosh, 2013). Most well-known DNA helicases involved in the maintenance of genome integrity are RecQ family helicases. Human RecQ helicases belong to SF2 helicase superfamily, share three highly conserved protein domains, and unwind a variety of DNA structures resembling DNA repair intermediates *in vitro* (Croteau et al., 2014). The RecQ helicases are Janus-faced. Genetic defects in these helicases are linked to diseases prone to develop cancers. However, targeted depletion of RecQ helicases in cancer cells decreased cell proliferation. It is interpreted that in the absence of functional RecQ helicases, genome instability increases which leads to the development of cancer. At the same time, upregulation of RecQ helicase is required to properly deal with lesions occurred during DNA replication in rapidly dividing cancer cells and, thus, it ensures the sustained growth of cancers (Brosh, 2013). RecQ-type helicases WRN and RecQL1 are expressed highly in several cancers and anticancer effects of siRNA against these genes, based on mouse models, were reported (Futami and Furuichi, 2015). Small-molecule inhibitors of helicase activities of WRN and another RecQ helicase BLM have been identified and both displayed anti-proliferative activity synergistically in the presence of chemotherapy drugs (Aggarwal et al., 2011, 2013; Nguyen et al., 2013).

Traditional anti-cancer drugs target DNA directly to trigger replication stress. Alkylating agents such as nitrogen mustard originated from chemical warfare during World Wars I and II, and their selectivity to kill cancer cells was dependent on the quantitative differences in the cell division rate between normal and cancer cells. Subsequently, numerous DNA-interacting agents were developed for the treatment of cancers including DNA crosslinkers, intercalators, and double-stranded DNA breaking agents (Hurley, 2002). The strategies to increase replication stress did not depend only on the DNA-interacting chemicals. Competitive inhibitors targeting enzymes required to maintain the pool of dNTPs are another type of conventional approaches to increase replication stress in cancer cells. Emerging approaches include (i) interfering with ATR-Chk1 replication checkpoint signaling to cause stalling and collapse of replication forks, (ii) inhibition of Wee1 kinases, that produces incompletely DNA-replicated cells, leading to mitotic catastrophe, and (iii) inhibition of histone deacetylases that dysregulates DNA replication (Conti et al., 2010; Puigvert et al., 2016; Zhang et al., 2016). DNA replication *per se* could be targeted by inhibiting enzymes responsible for the assembly of DNA replication complex and the initiation and elongation of DNA synthesis. These includes DDK and CDK kinases, proliferating cell nuclear antigen (PCNA), topoisomerases, DNA polymerases, and replicative helicase MCM (or CMG as an activated complex) (Berdis, 2008; Rodriguez-Acebes et al., 2010; Kang et al., 2012; Simon and Schwacha, 2014; Huggett et al., 2016; Roskoski, 2016).

Unwinding of duplex DNA during eukaryotic DNA replication is catalyzed by the CMG (Cdc45/Mcm2-7/GINS) helicase complex composed of three replication factors: Cdc45 protein, the Mcm2-7 and the GINS complexes (Moyer et al., 2006). Expression levels of Mcm subunits are down-regulated in differentiated somatic cells in keeping with its function in cell proliferation (Gupta and Brosh, 2008). On the other hand, enhanced expression of Mcm proteins have been reported in many cancer cells derived from patients (Neves and Kwok, 2017). The roles of the Mcm proteins in cancer progression have been linked to at least two cancer hallmarks including enhanced proliferation and regulation of replicative stress. For its catalytic role during DNA replication, the Mcm helicase has been considered as an emerging target for cancer therapy. The Mcm2-7 complex consists of six different Mcm ATPase subunits, however, the complexes in higher eukaryotes are not active as helicases (Kang et al., 2012). The Mcm2-7 complex is activated when it forms a macromolecular complex with Cdc45 and the GINS complex (Ilves et al., 2010). Until now, a few molecules have been identified to inhibit helicase activities of the budding yeast Mcm2-7, which is active as a helicase *in vitro* in certain conditions and the human Mcm4/6/7 subcomplex which is also active, but different from the Mcm2-7 complex in helicase activity (Simon and Schwacha, 2014). It appears that further screening of potential candidate compounds is required using the human CMG complex *in vitro*. Other approaches identified small molecules modulating the level of Mcm proteins in cells instead of inhibiting *in vitro* helicase activity. In this review, we discuss our current knowledge and progress on the development of chemotherapeutic drugs that target the human replicative helicase CMG and propose perspectives for the future of cancer treatment.

## CMG: mechanism and function in DNA replication

### DNA replication initiation and identification of replicative helicase CMG

The CMG helicase complex consists of 11 polypeptides. Its assembly is cell-cycle regulated and takes place on DNA in order to unwind duplex DNA during DNA replication. DNA replication occurs once per cell cycle, and the entire process is tightly regulated by multiple processes participated by dozens of proteins. The framework of current DNA replication model was first suggested by Jacob et al. (1963). In the replicon model, an initiator made from chromosome acts on a *cis*-acting replicator sequence allowing the beginning of the replication which spreads along the chromosome. Around 30,000–50,000 replicators, which we now call replication origin, are activated at each cell division in humans (Méchali, 2010).

The initiation of protein assembly reaction on DNA replication origins is relatively well understood in a budding yeast *Saccharomyces cerevisiae (S. cerevisiae)*. During late mitosis and G1-phase, the heterohexameric origin recognition complex (ORC) binds to the origin and recruits Cdc6 and Cdt1 proteins (Siddiqui et al., 2013). The resulting complex facilitates loading of two Mcm2-7 complexes onto the protein-origin DNA complex in a head-to-head fashion forming the pre-replication complex (pre-RC) and this assembly is also known as replication licensing (Riera et al., 2017). Loading of other replication factors onto chromatin is governed by the two S-phase specific kinases, DDK and CDK (Tanaka and Araki, 2013). Sld3-Sld7-Cdc45 binds to the origin during G1 and this step is dependent on phosphorylation of Mcm2-7 by DDK. Essential CDK phosphorylation occurs on Sld3 and Sld2, making them have affinity to Dpb11. Dpb11 is a part of the pre-loading complex (pre-LC) composed of Dpb11-Sld2-GINS-Polymerase (Pol) ε. Therefore, CDK phosphorylation leads to the assembly of a multi-protein complex onto the chromosome making the complex ready to initiate DNA replication and the complex in this state is called the pre-Initiation complex (pre-IC). These multi-step processes ultimately lead to the formation of the CMG helicase complex from Cdc45, the Mcm2-7, and the GINS and Mcm10 protein activates the CMG helicase to create replication forks (Kanke et al., 2012; van Deursen et al., 2012; Watase et al., 2012). Two CMGs converted from double hexameric Mcm2-7 move in opposite directions with polymerases (Pol ε, Pol δ, Pol α/primase) elongating new DNA chains (Riera et al., 2017). Humans have orthologues of the replication proteins described above except for Sld7 (MTBP is a functional homolog of Sld7) (Boos et al., 2013). However, the mechanism of loading of replication factors are not as clear as that of the budding yeast.

**Figure 1:**
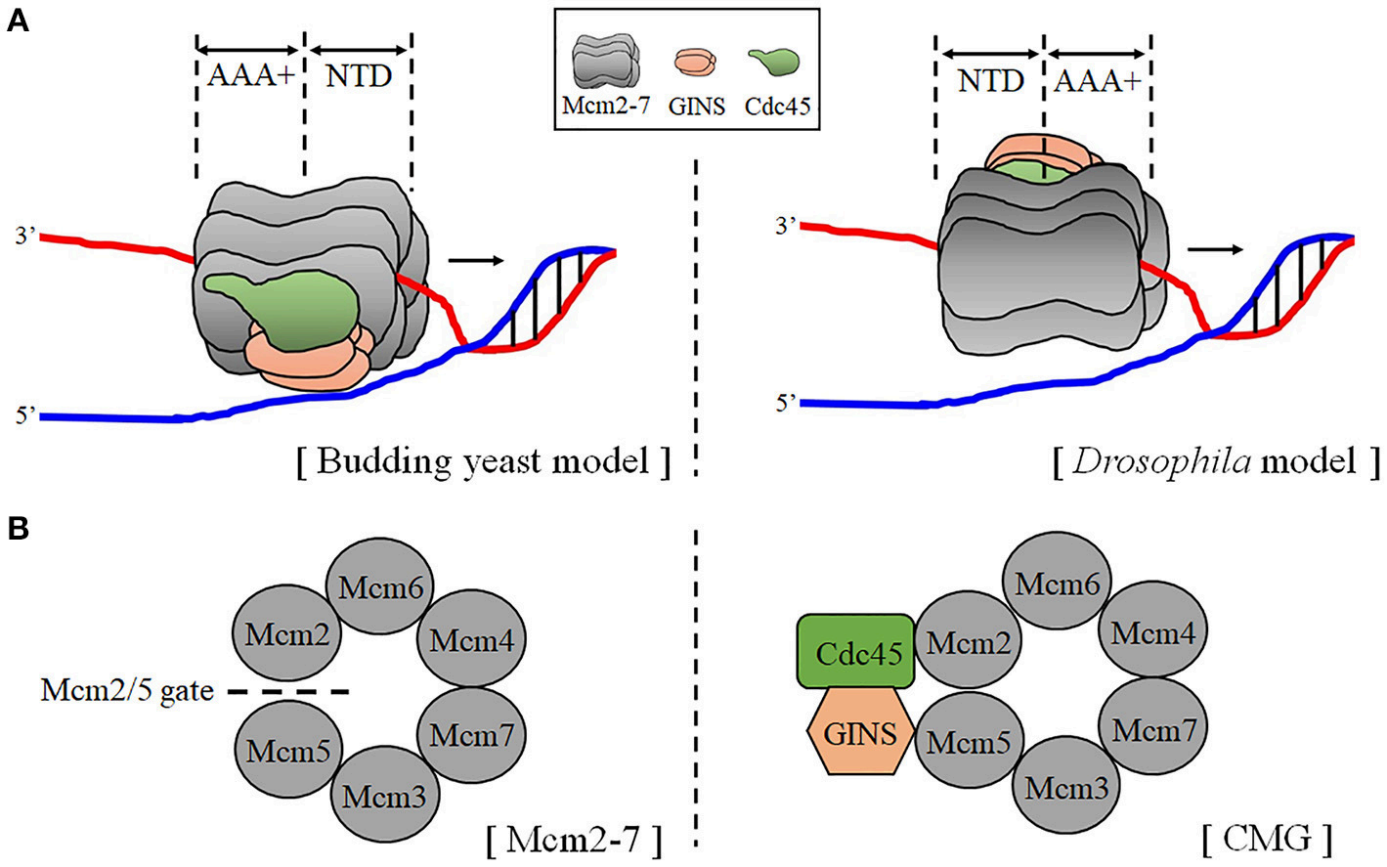
Schematic structure of the CMG helicase. **(A)** Two transloaction models of the CMG helicase on DNA. The orientations of the CMG with respect to translocation on DNA are illustrated based on the findings from cryo-EM studies of budding yeast (left) and the *Drosophila* (right) CMG. The N-tier faces dupelx DNA in the budding yeast model, while the C-tier (the AAA+ plus CTD domains) faces duplex DNA in the *Drosophila* model. **(B)** Structural comparison of the Mcm2-7 and the CMG complex. The views from the NTD of the Mcm2-7 (left) and the CMG (right) are presented. The Mcm2/5 gate is open as indicated in the Mcm2-7, while the gate is closed by bound Cdc45 and the GINS in the CMG. According to Costa et al. (2011), Cdc45-GINS binding to the Mcm2-7 complex surface bridges Mcm2 and 5 and the discontinuity between Mcm2 and Mcm5 is sealed by a nucleotide, which is omitted in the structure for clarity.

The six subunits (Mcm2, 3, 4, 5, 6, and 7) of the hexameric Mcm2-7 were discovered from genetic screenings of mutants defective in artificial minichromosome maintenance (Mcm) in attempts to isolate replication initiator proteins (Maine et al., 1984). All six members showed sequence similarity (20–30%) and shared a characteristic structural feature; AAA+ (ATPases associated with various cellular activities) domains flanked by the N- and C- terminal domains (NTD and CTD) (Tye, 1999; Parker et al., 2017). Six subunits are radially arranged in the hexameric Mcm2-7 complex, forming a central channel that DNA can access (Costa et al., 2011) (Figure 1B).

Cdc45 was originally identified as a cold-sensitive cell division cycle (cdc) mutant involved in the regulation of DNA replication and the *CDC45* gene was shown to have genetic interactions with *ORC2, MCM3, MCM5, MCM7* genes (Moir et al., 1982; Hardy, 1997). Chromatin loading of Sld3 and Cdc45 is mutually exclusive and experimental data suggested that phosphorylation of the Mcm2-7 by DDK enhances association of Sld3-Sld7-Cdc45 on chromatin in *S. cerevisiae* (Tanaka and Araki, 2013).

A subunit of the GINS was identified from the screening of synthetic lethal mutants with the temperature-sensitive *dpb11-1* allele in *S. cerevisiae* (Kamimura et al., 1998). One of the mutant isolated was named *sld5* (synthetic lethality with *dpb11-1 5*). Other three subunits of the GINS were identified from genetic screenings using *sld5* mutants and by immunoprecipitation assays, and they were named Psf (partner of Sld five) 1, Psf2, and Psf3 (Takayama et al., 2003). Independent of this, other screening efforts using temperature-induced degradation of target proteins and analyses of cell cycle progression defects also led to identification of the GINS subunits in *S. cerevisiae* (Kanemaki et al., 2003). Homologs of the yeast GINS were also identified in *Xenopus* and the ring-like structure of the four-subunit GINS was characterized (Kubota et al., 2003). The GINS is recruited to DNA in the CDK-dependent manner in S-phase as a part of pre-LC as described above.

Since identification of Mcms as factors required for replication licensing, their molecular functions at the chromatin have been investigated. The presence of ATPase motifs in the central domain of all Mcms, inhibition of DNA replication initiation in cells by nuclear microinjection of anti-human Mcm2 antibodies, and the presence of the stable complex containing all six Mcm subunits in cells raised the possibility that the Mcm complex is the replicative helicase that is responsible for progression of replication forks to generate template DNA during DNA replication (Todorov et al., 1994; Ishimi et al., 1996; Schulte et al., 1996). First evidence came from studies with human proteins. The human Mcm complexes were isolated from HeLa cells and it was found that the 3′–5′ DNA helicase activity was intrinsically associated with the non-canonical Mcm complex containing only Mcm4, 6, and 7 (Ishimi, 1997). Subsequently, it was shown that mouse Mcm4/6/7 complex reconstituted by baculovirus infection has intrinsic helicase activity (You et al., 1999). Fission yeast Mcm4/6/7 was shown to have a similar activity, while the hexameric Mcm2-7 complexes had not been found to have DNA helicase activity (Lee and Hurwitz, 2000). However, the predominant form of Mcm complex present in *Xenopus* egg extracts is a heterohexamer containing all six subunits and only the Mcm2-7 complex supported DNA replication in the extract, although *Xenopus* Mcm4/6/7, not Mcm2-7, has intrinsic helicase activity (Prokhorova and Blow, 2000; Ying and Gautier, 2005). Besides, the ATPase activity of the *Xenopus* Mcm2-7 is required for DNA unwinding (Ying and Gautier, 2005). Moreover, all Mcm2-7 proteins are essential for the elongation of chromosome replication (Labib et al., 2000), indicating that heterohexameric Mcm2-7 is the *in vivo* replicative helicase. Until now, only *S. cerevisiae* Mcm2-7 has been shown to have DNA helicase activity comparable to that of Mcm4/6/7 under certain conditions in a manner dependent on specific anions (Bochman and Schwacha, 2008). For these and other reasons as discussed below, screenings of inhibitor molecules targeting the Mcm complexes have been performed only with Mcm4/6/7 and *S. cerevisiae* Mcm2-7.

Cdc45 was determined as a factor required for DNA unwinding in *Xenopus* egg extract and was shown to physically interact with Mcm proteins (Mimura et al., 2000; Walter and Newport, 2000). Neutralizing antibodies against Cdc45 abolished chromosomal unwinding (Pacek and Walter, 2004). Furthermore, DNA helicase activity was detected with both anti-Mcm and anti-Cdc45 immunoprecipitates from chromatin replication reactions in *Xenopus* egg extracts and the complex containing Mcm2-7 and Cdc45 was partially purified, suggesting the possibility of activation of Mcm2-7 helicase activity by Cdc45 (Masuda et al., 2003). Finally, Moyer et al. (2006) isolated a high-molecular-weight complex containing Cdc45 from *Drosophila* embryo extracts, establishing that the eleven-subunit complex containing Mcm2-7, GINS, and Cdc45 (CMG) is an active helicase. The same group reconstituted the *Drosophila* CMG complex using purified proteins from the baculovirus expression system, and showed that the Mcm2-7 is activated by association with Cdc45 and the GINS (Ilves et al., 2010). Human CMG was also reconstituted similarly as described for *Drosophila* CMG, and *S. cerevisiae* CMG complex was isolated from yeast cells overexpressing all 11 subunits (Kang et al., 2012; Georgescu et al., 2014). All the CMG complexes purified up to date displayed 3′–5′ DNA helicase activities.

### Structure of the CMG complex

The CMG complex is the core of the replication protein complex and its near-atomic three-dimensional structure was revealed through the cryo-EM and X-ray crystallography of the sub-complexes. Overall structure of the Mcm2-7 is a toroid shaped by a regular arrangement of the six Mcm monomers (Costa et al., 2011) (Figure 1A). All six Mcm protomers display the same domain organization, suggesting they are derived from one ancestral gene and this is supported by the presence of homo-oligomeric Mcm complexes in archaea (Abid Ali and Costa, 2016). The N-terminal domain (NTD) of Mcm can be further divided into several subdomains; (i) a helical domain A involved in the DDK-mediated conformational switch and the activation Mcm helicase in archaea. This domain contains an oligonucleotide-binding (OB)-fold which makes contact with ssDNA and is required for Mcm oligomerization and the loading of the Mcm2-7 to replication origins (Fletcher et al., 2003; Slaymaker and Chen, 2012; Froelich et al., 2014). (ii) A Zinc (Zn)-finger domain, along with the yeast-specific Mcm5-7 inter-ring contact, provides an interface for the Mcm2-7 double hexamer formation on the chromatin (Li et al., 2015). (iii) Eukaryotic Mcms have N-terminal extensions (NTEs), which are especially long in Mcm2, Mcm4, and Mcm6 (Riera et al., 2017). These extensions play roles in the DDK-dependent regulation of replication initiation. In contrast, archaeal Mcms lacks the NTEs.

The AAA+ ATPase domain of Mcms consists of Walker A and B motifs which associates with an arginine finger from the adjacent subunit for its ability to hydrolyze ATP (Davey et al., 2003). Inter-subunit interactions in the ATPase domain requires four conserved hairpins, more specifically, loops from two adjacent subunits including the allosteric communication loop (ACL) and helix-2-insert (H2I) of the first subunit, and H2I and the pre-sensor1 (PS1) hairpin of the second subunit (Li et al., 2015). PS1 projects into the central cavity of the Mcm2-7 and is involved in helicase translocation (Miller et al., 2014; Petojevic et al., 2015). H2I loops from all six Mcm AAA+ modules form a right-handed spiral staircase in the channel of the Mcm2-7 and are suggested to be involved in the initial melting of origin DNA (Li et al., 2015). The atomic model of CMG-ssDNA (14-mer) obtained from cryo-EM revealed that six out of the 14 nucleotides interact with the OB loops of Mcm4 and Mcm7 in the N-tier ring, whereas remaining 8 nucleotides form a right-handed B-form spiral structure in the C-tier ring that interacts with PS1 and H2I loops of Mcm3, 5, 2, and 6 (Georgescu et al., 2017). The right-handed spiral staircase of H2I pore loops are also present in other hexameric helicases (Abid Ali and Costa, 2016). In the structure of ssDNA bound-E1 AAA+ domain of bovine papillomavirus, the ATP-bound active site of E1 has a pore loop on top of the spiral staircase, while the ATP-free site has the loop at the bottom. This observation had led to the suggestion of the rotary translocation mechanism of hexameric DNA helicases.

The CTD domain of Mcm adopts a winged-helix (WH) domain which is flexibly tethered to the AAA+ domain (Parker et al., 2017). The WH domain in the archaeal Mcm controls ATPase activity of Mcms allosterically (Wiedemann et al., 2015). WH domains of eukaryotic Mcm3 and Mcm6 have been shown to be required for pre-RC assembly through interactions with Cdc6 and Cdt1, respectively (Liu et al., 2012; Frigola et al., 2013). In the CMG complex, unlike in the Mcm2-7, the WH domains Mcm5 and Mcm6 are stacked inside the central channel restricting the diameter of the channel as discussed below (Yuan et al., 2016).

Cryo-EM analyses of a single Mcm2-7 complex have revealed it has spiral cracked-ring structure containing a gap between Mcm2 and Mcm5 (Costa et al., 2011; Lyubimov et al., 2012; Figure 1B). The cryo-EM analysis of the Cdt1-Mcm2-7 heptameric complex of *S. cerevisiae* revealed that Cdt1 wraps around NTDs of Mcm2, Mcm4, and Mcm6, contributing to stabilization of the complex (Yuan et al., 2017; Zhai et al., 2017). Interactions between Cdt1 and the three Mcms were also observed by cross-linking experiments (Frigola et al., 2017). The role of the Cdt1 suggested in the complex assembly was that it acts as a “brace” which maintains the open spiral structure of Mcm2-7 until ATP hydrolysis (induced by ORC-Cdc6-Cdt1-Mcm2-7 on DNA) triggers the Mcm2-5 gate to close, which in turn leads to the release of Cdt1. In keeping with this, another report also showed that ATP hydrolysis by Mcm2-7, which is coupled to the ring closure, releases the associated Cdt1 protein (Ticau et al., 2017). In the CMG structure, Cdc45 and the GINS complex close the Mcm2-5 gate through binding at the side of the Mcm2-7, forming ATPase site essential for translocation on DNA (Abid Ali and Costa, 2016; Figure 1B). Further work is needed to unveil switching mechanism from double-hexameric Mcm2-7 to two single CMG complexes. The orientation of the CMG with respect to translocation on DNA was analyzed by cryo-EM of the CMG on a model DNA. However, studies on *S. cerevisiae* and *Drosophila* CMG gave rise to different results (Costa et al., 2014; Georgescu et al., 2017; Figure 1A). The *S. cerevisiae* CMG had an N to C-tier polarity of translocation which is opposite to the model described based on *Drosophila* CMG studies.

### Unwinding mechanism of the CMG helicase

The authors of the paper above (Georgescu et al., 2017) proposed an interesting model of the initial melting of the origin by the CMG. The N-termini of the two Mcm2-7 complexes are facing each other in the double hexameric state (head-to-head), encircling dsDNA (Figure 1A). If the CMG complex has a N-tier to C-tier polarity in translocation, they must pass each other and it requires them to encircle opposite strands of the DNA. If this is the case, it is imperative that each CMG encircles ssDNA after activation. The cryo-EM structure of the double hexameric Mcm2-7 bound to DNA gave another insight into the initial melting of DNA. Based on the structure of yeast double-hexmeric Mcm2-7, two Zn-finger domains from each hexamer form two smaller rings, but large enough to accommodate duplex DNA in the interface of hexamers (Li et al., 2015; Abid Ali and Costa, 2016). However, two rings are not co-axial and even are twisted with respected to each other. This would make distortion of DNA resulting in the opening of the origin. The structure of double hexameric MCM2-7 bound to duplex DNA was analyzed by another group and the authors suggested that a lateral shift and tilt of the N-tier rings would help separate and extrude lagging strands through the Mcm2-Mcm5 gates (Noguchi et al., 2017).

Currently, two proposals are available to account for the mode of translocation of hexameric helicases on DNA (Costa and Onesti, 2009). According to the “strand extrusion (or side channel extrusion)” model, the CMG translocates encircling dsDNA and the unwound strand is extruded via a side channel formed between NTD and the AAA+ domain of the Mcm2-7. In the “steric exclusion” model, the CMG encircles only the leading-strand template while the lagging-strand template is excluded from the interior of the complex. The latter model is supported by biochemical data obtained by others (Langston and O'Donnell, 2017; Trakselis et al., 2017). The inside channel of the Mcm2-7 in the CMG was shown to be large enough to accommodate dsDNA (Costa et al., 2011; Sun et al., 2015). In contrast, it was found that the WH domain of Mcm5 protrudes into the interior axial channel of the CTD ring, rendering the pore size too narrow for dsDNA accommodation, and this finding was used as evidence to support the steric exclusion model (Yuan et al., 2016). However, unwinding studies have shown that CMG is able to move along the duplex DNA, suggesting that dsDNA can be accommodated in the central channel of the CMG (Kang et al., 2012). The steric exclusion model is supported by the results obtained from experiments using *Xenopus* egg extracts and DNA substrates containing roadblocks on selected DNA strands. The CMG stalled when the leading strand had obstructions, but did not when obstructions were in the lagging strand, suggesting that the lagging strand is excluded from the interior of the CMG (Fu et al., 2011). Similar *in vitro* experiments were performed with *S. cerevisiae* CMG. However, blocks on either strand resulted in inhibition of CMG unwinding, which made authors propose a modified steric exclusion model in which both strands enter the channel and the duplex unwinding occurs internally, followed by exclusion of the non-tracking strand (Langston and O'Donnell, 2017). And Botchan's group performed cross-linking of CMG with fork-structured DNA, revealing interactions of the leading strand with the inside channel while lagging strand contacts the Mcm2-7 external surface (Petojevic et al., 2015). The same study has led to a proposal that Cdc45 acts as a guardian of the Mcm2-Mcm5 gate which captures the leading strand in a 3′->5′ orientation.

### Reconstitution of the CMG activation and DNA replication reaction

Identification of replication factors, their interactions, and enzymatic functions allowed researchers to reconstitute DNA replication *in vitro*. Formation of active CMG in eukaryotes depends on multiple stepwise reactions that can be largely segregated into two stages as described above; (i) formation of the two Mcm2-7 on an origin and (ii) conversion of the double hexameric Mcm2-7 into two single CMG complexes.

Through the single molecule analysis using fluorescence tagged proteins, sequential loading of two hexameric Mcm2-7s on DNA was monitored (Ticau et al., 2015). The first Mcm2-7 was recruited by the ORC/Cdc6 followed by the recruitment of the second Mcm2-7 by the first Mcm2-7. Although this study proposed that single ORC is responsible for the loading of double hexameric Mcmc2-7s, the study published recently suggested that two ORC molecules are required for the loading of two Mcm2-7s (Coster and Diffley, 2017). The study has shown that relative orientation of the ORC binding sites was critical for the bidirectional loading of two Mcm2-7 molecules.

The *S. cereviseae* CMG was assembled with purified components and its assembly and activation was indirectly monitored by polymerase reactions (Yeeles et al., 2015). The *in vitro* replication initiation system has allowed detailed analyses of the CMG activation and functional interactions between the CMG and other replication factors (Deegan et al., 2016; Douglas and Diffley, 2016; Devbhandari et al., 2017; Frigola et al., 2017; Gan et al., 2017; Kurat et al., 2017; Looke et al., 2017; Yeeles et al., 2017). At the same time, incubation of purified *S. cereviseae* CMG with other factors such as polymerases and the scaffolding factor Ctf4 has made possible microscopic analysis of the minimal replisome and studies on the distribution of DNA polymerases on leading and lagging strands (Georgescu et al., 2014, 2015, 2017; Sun et al., 2015). Reconstitution of the CMG helicase assembly reaction *in vitro* can provide a platform to screen and test candidate molecules that could inhibit the CMG formation.

## The CMG as a potential target for cancer therapy

### The CMG and cancer

The correlation between Mcm and cancer was first proposed 20 years ago by an observation of increased levels of nuclear Mcm7-positive cells in the malignant form of cutaneous keratinocytic tumors. This was further supported by subsequent studies that reported elevated levels of other Mcms (Mcm2 and Mcm5) in various types of cancers (Hiraiwa et al., 1997; Todorov et al., 1998; Freeman et al., 1999).

### The CMG and genome instability

There is an excess amount of the Mcm2-7 on chromatin, playing a role in maintaining genome stability, particularly under replicative stress by activating dormant origins as backups during DNA replication (Ge et al., 2007; Ibarra et al., 2008). In accordance and compatible with this, some types of cancer are also caused by low activity of Mcms. A genetic screen for spontaneous chromosomal instability in mice using *N*-ethyl-*N*-nitrosourea (ENU) mutagenesis followed by genetic mapping and sequencing identified a mutant allele of Mcm4 (Shima et al., 2003, 2007). The mutated Mcm4 (F345I) (*Mcm4*^*Chaos*3^) in mice caused reduction in overall levels of Mcms, leading to high incidences of mammary adenocarcinomas (Shima et al., 2007). The phenylalanine at 345 in Mcm4 is well conserved, located downstream from a Zn-finer domain, and important for its interaction with other Mcms. The mutation at the corresponding residue of the budding yeast Mcm4 displayed a minichromosome loss phenotype. Mouse embryonic fibroblast cells (MEFs) with the Mcm4 (F345I) allele were more susceptible to chromosome breakage under replicative stress. One other group reported that this allele caused instability of Mcm2-7 without affecting helicase activity of the CMG *in vitro* (Kawabata et al., 2011). The same group also showed increases in dormant origin activation in *Mcm4*^*Chaos*3/*Chaos*3^ MEFs and cells are arrested in M phase with increasing numbers of abnormal chromosomes. These results suggest that the impaired ability of cells under replication stress to activate the dormant origins leads to the loss of tumor suppression. A dominant allele of mouse Mcm4 (D573H) enhanced genome instability leading to increased incidence of cancer development (Bagley et al., 2012). Unlike the Mcm4 (F345I) allele, this mutant did not show any detectable change protein stability. However, it appears that Mcm4 (D573H) assemble into the Mcm2-7, leading to formation of the inactive holocomplex. The mutation at the corresponding residue of budding yeast Mcm4 did not support growths of mutant cells. When the expression levels of Mcm2 were reduced to one-third of wild-type levels, the average life span of mice decreased markedly due to high incidence of cancers, and the majority of them were T- and B-cell lymphoma (Pruitt et al., 2007). Decreased expression of Mcm2 led to elevated levels of Ser139 phosphorylated histone H2AX (γ-H2AX) foci in cultured muscle satellite cells, which is indicative of thigh incidence double-strand DNA breaks. These phenotypes are consistent with the proposed function of dormant origins as backups during DNA replication which would prevent the incidence of double-stand DNA breaks that could arise from stalled DNA replication forks.

### The CMG as a prognostic marker for cancers and a target for cancer therapy

Most of the Mcm-related cancers have shown positive correlation between Mcm expression levels and the malignancy of cancers in keeping with the intrinsic proliferative function of Mcms. For example, Mcms were abnormally highly up-regulated in pancreatic cancer and gliomas, and this was closely related to rapid cancer progression (Hua et al., 2014; Peng Y. P. et al., 2016). The proliferative index of Mcm2 and Mcm3 can be used as a prognostic marker for survival of patients with small intestinal neuroendocrine neoplasm (SI-NEN) (Schimmack et al., 2016). The cancer types where increased levels of the CMG components were observed are summarized in the Table 1.

**Table 1:** Overexpression of the CMG components in cancers.

**Subunit**	**Cancer types**	**References**
Mcm2	Adrenocortical dysplasia Anal carcinoma Breast carcinoma B-cell lymphoma Cervical cancer Colon cancer Colorectal cancer Dysplastic squamous oesophageal epithelium and Barrett's mucosa Esophageal squamous cell carcinoma Gastric adenocarcinoma Glioma Laryngeal squamous cell carcinoma Meningioma Merkel cell carcinoma Muscle-invasive urothelial cancer Non-small cell lung cancer Oral squamocellular carcinoma Pancreatic cancer Pancreaticobiliary adenocarcinoma Prosate cancer Renal cell carcinoma Salivary gland tumors Small intestinal neuroendocrine neoplasm Vulval intraepithelial neoplasia	Szajerka et al., 2008 Scapulatempo-Neto et al., 2017 Gonzalez et al., 2003; Wojnar et al., 2011 Obermann et al., 2005 Ishimi et al., 2003 Giaginis et al., 2009 Guzinska-Ustymowicz et al., 2008 Going et al., 2002 Kato et al., 2003 Tokuyasu et al., 2008; Giaginis et al., 2011 Hua et al., 2014 Chatrath et al., 2003 Saydam et al., 2010 Gambichler et al., 2009 Korkolopoulou et al., 2005 Yang et al., 2006 Szelachowska et al., 2006; Razavi et al., 2015 Peng Y. P. et al., 2016 Abe et al., 2016 Meng et al., 2001 Rodins et al., 2002 Vargas et al., 2008 Schimmack et al., 2016 Davidson et al., 2003
Mcm3	Breast cancer Cervical cancer Dysplastic squamous oesophageal epithelium and Barrett's mucosa Glioma Meningioma Ovarian cancer Papillary thyroid carcinoma Salivery gland epithelial tumor Small intestinal neuroendocrine neoplasm	Kwok et al., 2015 Ishimi et al., 2003 Going et al., 2002 Hua et al., 2014 Saydam et al., 2010 Kobierzycki et al., 2013 Igci et al., 2014 Zielinski et al., 2016 Schimmack et al., 2016
Mcm4	Breast cancer Cervial cancer Esophageal cancer Meningioma Merkel cell carcinoma Non-small cell lung cancer Pancreatic cancer	Kwok et al., 2015 Ishimi et al., 2003 Huang et al., 2005; Choy et al., 2016 Saydam et al., 2010 Gambichler et al., 2009 Kikuchi et al., 2011 Peng Y. P. et al., 2016
Mcm5	Breast cancer Cervical dysplasia (or cancer) Colon carcinoma Bladder carcinoma Esophageal cancer Gastric adenocarcinoma Meningioma Prosate cancer Urothelical cancer	Kwok et al., 2015 Ishimi et al., 2003; Murphy et al., 2005 Freeman et al., 1999 Freeman et al., 1999 Williams et al., 2004 Giaginis et al., 2011 Saydam et al., 2010 Dudderidge et al., 2010 Stoeber et al., 1999; Korkolopoulou et al., 2005
Mcm6	Breast cancer Cervical cancer Mantle cell lymphoma Pancreatic cancer	Kwok et al., 2015 Ishimi et al., 2003; Henderson et al., 2011 Schrader et al., 2005 Peng Y. P. et al., 2016
Mcm7	Acute myeloid leukemia Breast cancer Cervical cancer Cutaneous keratinocytic tumor Endometrial carcinoma Glioma Head and neck squamous cell carcinoma Meningioma Merkel cell carcinoma Mesothelioma Neuroblastoma Oral squamous cell carcinoma Prostate cancer Small lung adenocarnoma	Lee et al., 2017 Kwok et al., 2015 Ishimi et al., 2003; Henderson et al., 2011 Hiraiwa et al., 1997 Li et al., 2005 Facoetti et al., 2006a Cromer et al., 2004 Saydam et al., 2010 Gambichler et al., 2009 Kimura et al., 2009, 2013 Shohet et al., 2002 Tamura et al., 2010 Padmanabhan et al., 2004 Fujioka et al., 2009
Psf1	Breast cancer Hepatocellular carcinoma Lung cancer Non-small cell lung cancer Prostate cancer	Nakahara et al., 2010 Zhou et al., 2015 Zhang J. et al., 2015 Kanzaki et al., 2016 Tahara et al., 2015
Psf2	Breast cancer Cervical cancer Intrahepatic cholangiocarcinoma	Peng L. et al., 2016 Ouyang et al., 2017 Obama et al., 2005
Psf3	Colon carcinoma Colorectal cancer Non-small cell lung carcinoma Lung adenocarcinoma	Nagahama et al., 2010a Sun et al., 2014 Tane et al., 2015 Hokka et al., 2013; Tauchi et al., 2016
Sld5	Bladder cancer Breast cancer Cervical cancer Colon cancer Gastric cancer Liver cancer Lung cancer Prostate cacer	Yamane et al., 2016 Mohri et al., 2013 Mohri et al., 2013 Mohri et al., 2013 Mohri et al., 2013 Mohri et al., 2013 Mohri et al., 2013 Mohri et al., 2013
Cdc45	Acute lymphoblastic leukemia Acute promyelocytic leukemia Acute T-cell leukemia Breast carcinoma Cervical cancer Chronic myelogenous leukemia Glioblastoma Histiocytic leukemia Lung cancer Osteosarcoma Papillary thyroid cancer Tongue squamous cell carcinoma	Pollok et al., 2007 Pollok et al., 2007 Pollok et al., 2007 Pollok et al., 2007 Di Paola and Zannis-Hadjopoulos, 2012 Pollok et al., 2007 Pollok et al., 2007 Pollok et al., 2007 Tomita et al., 2011 Pollok et al., 2007 Sun et al., 2017 Li et al., 2008

Mcm2 is extensively studied as a prognostic marker for cancer. Increased levels of Mcm2 expression have been detected in a variety of cancers (Table 1), and it is often accompanied with overexpression of Ki-67, a representative marker for cell proliferation (Meng et al., 2001; Going et al., 2002; Chatrath et al., 2003; Davidson et al., 2003; Yang et al., 2006; Guzinska-Ustymowicz et al., 2008, 2009; Szajerka et al., 2008; Vargas et al., 2008; Giaginis et al., 2009; Razavi et al., 2015; Scapulatempo-Neto et al., 2017). It has been shown that Mcm2 is a prognostic marker superior to Ki-67 in oral cavity squamocellular carcinoma and kidney tumor samples (Rodins et al., 2002; Szelachowska et al., 2006). The validity of Mcm2 as a prognostic marker is also dependent on the genetic background in gastric adenocarcinoma (Tokuyasu et al., 2008; Giaginis et al., 2010). Immunocytochemistry of Mcm2, together with p53, when added to the conventional cytological evaluation, increased sensitivity in the diagnosis of pancreaticobiliary adenocarcinoma (Abe et al., 2016). It has been shown that Mcm2 expression has positive correlation with malignancy grade in breast carcinoma and esophageal squamous cell carcinoma (Gonzalez et al., 2003; Kato et al., 2003; Wojnar et al., 2011). Association of Mcm2 with clinicopathological characteristics was also observed in other types of cancer including diffuse large B-cell lymphoma and muscle-invasive urothelial cancer (Korkolopoulou et al., 2005; Obermann et al., 2005).

Overexpression of Mcm3 was also detected in many cancers, for example, salivary gland epithelial tumors and papillary thyroid carcinoma (Igci et al., 2014; Zielinski et al., 2016). There was positive correlation between levels of Mcm3 expression and malignancy grade in ovarian cancers (Kobierzycki et al., 2013). Increased levels of Mcm3, along with Mcm2 and Mcm7, have shown poor prognosis (Hua et al., 2014).

Mcm4 expression also increased in diverse cancers. Elevated levels of Mcm4 were detected in non-small cell lung cancer (NSCLC) compared to neighboring normal bronchial epithelial cells (Kikuchi et al., 2011). Expression of Mcm4, along with Mcm2 and Mcm7, has significantly increased in Merkel cell carcinoma (Gambichler et al., 2009). It appears that levels of Mcm4 is closely associated with pathological grade of esophageal cancer, and its overexpression has been shown to correlate with lymph node metastasis and poor survival in adenocarcinoma patients (Huang et al., 2005; Choy et al., 2016).

The fraction of Mcm5-immunostained cells can be used to determine the pathological grade in urothelial cancers (Stoeber et al., 1999). Detection of elevated levels of Mcm5 in gastric aspirates and in urine sediments was effective in the prediction of esophageal and prostate cancer, respectively, with high sensitivity and specificity (Williams et al., 2004; Dudderidge et al., 2010). Mcm5 expression was significantly elevated in both HPV (human papilloma virus) dependent and independent cervical dysplasia (Murphy et al., 2005). Levels of Mcm5 expression is tightly associated with clinicopathological characteristics and patient survival in gastric adenocarcinoma and muscle-invasive urothelial cancer (Korkolopoulou et al., 2005; Giaginis et al., 2011).

Comparative analyses revealed that Mcm6 is a marker far superior to Ki-67 in predicting the outcome in mantle cell lymphoma (Schrader et al., 2005). Immunological assays using anti-Mcm6 and anti-Mcm7 antibodies were developed to efficiently detect cervical cancer (Henderson et al., 2011).

Expression levels of Mcm7 were also upregulated in a variety of cancer cells (Table 1; Freeman et al., 1999; Shohet et al., 2002; Cromer et al., 2004; Padmanabhan et al., 2004; Facoetti et al., 2006a). Conditional expression of Mcm7 in mice was shown to induce tumor formation (Yoshida and Inoue, 2003; Honeycutt et al., 2006). Mcm7, together with other cell proliferation markers, were used for differential diagnosis of reactive mesothelial cells and malignant mesothelioma cells (Kimura et al., 2009, 2013). The labeling index (LI) of Mcm7 in immunohistochemistry was more reliable than that of Ki-67 in several types of cancer and is strongly correlated to the tumor aggressiveness in astrocytoma and oral squamous cell carcinoma (Li et al., 2005; Facoetti et al., 2006b; Fujioka et al., 2009; Tamura et al., 2010; Choy et al., 2016). Mcm7 polymorphisms were shown to have a close relationship with relapse of acute myeloid leukemia (Lee et al., 2017). Mcm7, however, was not useful as a prognostic marker in colorectal cancer and as a risk factor for recurrence-free survival in patients with Duckes C colorectal cancer (Nishihara et al., 2008; Ishibashi et al., 2014).

It was shown that concurrent overexpression of all six Mcm subunits, rather than single individual Mcm subunits, is strongly correlated with poor survival in breast cancer patients (Kwok et al., 2015). It has been reported that expression of all Mcm2-7 subunits could lead to significant increases in meningiomas compared to arachnoidal tissue controls (Saydam et al., 2010). Oncogenic mutant p53 (mtp53) influenced chromatin enrichment of pre-RC components and protein-protein interactions were detected between mtp53 and Mcm subunits in triple negative breast cancer (TNBC) cells (Qiu et al., 2017). Enhanced expression of Mcms in total cellular proteins and in the chromatin-bound fractions were observed in cervical cancer cells (Ishimi et al., 2003). Immunocytochemistry for Mcm proteins has been shown to be more rapid and reliable than the traditional Papanicolaou test for cervical cancer (Mukherjee et al., 2007).

Overexpression of the GINS subunits were also reported in cancers. Psf1 was relatively highly expressed in breast tumor and lung cancer cells (Nakahara et al., 2010; Zhang J. et al., 2015). An increased ability of cancer cells to proliferate by Psf1 overexpression was observed by a mouse xenograft model (Nagahama et al., 2010b). Psf1 expression was found to be an independent prognostic marker for poor survival in NSCLC patients treated with surgery after preoperative chemotherapy or chemoradiotherapy (Kanzaki et al., 2016). Increased expression of Psf1 was associated with aggressiveness of hepatocellular carcinoma and prostate cancer (Tahara et al., 2015; Zhou et al., 2015). Overexpression of Psf2 was detected in intrahepatic cholangiocarcinoma, and tumorigenesis was promoted by elevated expression of Psf2 in early-stage cervical cancer (Obama et al., 2005; Ouyang et al., 2017). Interestingly, Psf2 knockdown in TNBC cells downregulated expression of matrix metallopeptidase 9 which is required for tumor invasion, thereby inhibited the migration and invasion of TNBC cells (Peng L. et al., 2016). However, it is unclear whether this regulatory role is CMG-related or independent of the Psf2 function. Psf3 expression was also increased in colon carcinoma compared to the neighboring normal mucosa (Nagahama et al., 2010a). The survival rate of the patient group with increased Psf3 expression was considerably lower than that of the reduced Psf3 expression group in NSCLC, lung adenocarcinoma, and colorectal cancer patients (Hokka et al., 2013; Sun et al., 2014; Tane et al., 2015; Tauchi et al., 2016). Finally, robust expression of Sld5 was detected from bladder cancer tissues and many cancer cell lines (Mohri et al., 2013; Yamane et al., 2016).

The level of the Cdc45 protein was upregulated in a variety of cancer-derived cell lines including carcinoma, sarcoma, leukemia, and lymphoma (Pollok et al., 2007; Tomita et al., 2011; Di Paola and Zannis-Hadjopoulos, 2012). The overexpression of Cdc45 closely correlated with tumor sizes and stages in papillary thyroid cancer (Sun et al., 2017). The expression of Cdc45 increased in tongue squamous cell carcinomas and the level of Cdc45 was shown to have positive correlation with grades of precancerous lesions in epithelial dysplasia (Li et al., 2008). Cdc45 overexpression phenocopied the proto-oncogne Myc-dependent phenotypes (Srinivasan et al., 2013). Thus, it was suggested that Cdc45 acts as a downstream effector of Myc-induced replication stress which is required for oncogenesis.

These results suggest that the proliferative function of CMG is generally required for the growth of tumors and, but to varying extents, depending on the type of cancer. Besides, overexpressions of CMG subunits are closely associated with malignancy. Therefore, CMG can act as not only a marker for cancer progression but also as a druggable target to treat cancers.

## Strategies to target the CMG for anticancer therapy

### Background for targeting the CMG for cancer therapy

Two theoretic models provide a rational basis to use Mcms as a target for treatment of cancers (Neves and Kwok, 2017). One model is involved with downregulation of Mcm in the hope of reducing tumor growth because sustained and elevated expression of Mcms would be essential for rapid proliferation of cancer cells. Consistent with this, knockdown of Mcm components by siRNA suppressed proliferation of cancer cells (Lau et al., 2010; Kikuchi et al., 2011; Toyokawa et al., 2011; Zhang X. et al., 2015). In addition, this holds true for Cdc45 and the GINS (Nakahara et al., 2010; Tane et al., 2015; Zhang J. et al., 2015; Sun et al., 2017). The precise mechanisms by which cancer cell proliferation is inhibited by depletion of the CMG components are still remains to be investigated. The siRNA-mediated knockdown of Mcm2, Mcm3, and Mcm7 inhibited medulloblastoma cell growth (Lau et al., 2010). However, the point of cell cycle arrest was not the same. For example, knockdown of Mcm3 resulted in G1 arrest with reduced levels of cyclin A, while knockdown of Mcm2 or Mcm7 resulted in G2/M arrest without any detectable change of cyclin A levels. Therefore, it is inappropriate to simply attribute the growth inhibitory effect by Mcm downregulation to low amounts of functional replicative helicase.

The other model focuses on excess amounts of Mcms which is important for cancer cells to survive, with maintaining minimal genome stability, in the presence of high degree of replicative stress (Neves and Kwok, 2017). Reduction of Mcms to limited extents does not impair DNA replication in normal condition (Lei et al., 1996; Crevel et al., 2007; Ibarra et al., 2008). However, cells became highly sensitive to chemicals that interfere with DNA replication in the presence of low amounts of Mcms because of limited activation of backup origins (Woodward et al., 2006; Ge et al., 2007; Ibarra et al., 2008; Chuang et al., 2010).

Based on the two primary mechanisms described above, several strategies have been explored to inhibit the CMG functions in order to develop anti-cancer therapeutics. The molecules targeting Mcms are listed and summarized in Table 2.

**Table 2:** Summary of small molecules targeting Mcms.

**Name**	**Classification**	**Targeting mechanism**	**References**
Genistein	Isoflavone	Downregulation of Mcm2	Majid et al., 2010
Trichostatin A	HDAC inhibitor	Downregulation of Mcm2 (through the activation of JNK signaling pathway)	Majid et al., 2010; Liu et al., 2013
Widdrol	Aromatic compund	Downregulation of Mcm2-7 (through the activation of RB by DNA damage)	Hong et al., 2009; Kwon et al., 2010; Yun et al., 2012
Lovastatin	HMG-CoA reductase inhibitor	Downreguation of Mcm2 (through the activation of JNK signaling pathway)	Zhang X. et al., 2015
Metformin	Biguanide	Downregulation of Mcm2-7	Kim et al., 2017
BETi	Molecular mimicker of acetylated histone	Downregulation of Mcm5	Mio et al., 2016
Breviscapine	Flavonoid	Downregulation of Mcm7	Guan et al., 2017
Heliquinomycin	Antibiotic compound	Inhibition of Mcm4/6/7 helicase	Ishimi et al., 2009; Toyokawa et al., 2011
Ciprofloxacin	Fluoroquinolone	Inhibition of Mcm2-7 and Mcm4/6/7 helicase	Simon et al., 2013

### Strategy I. downregulation of constituents of the CMG complex

**Figure 2:**
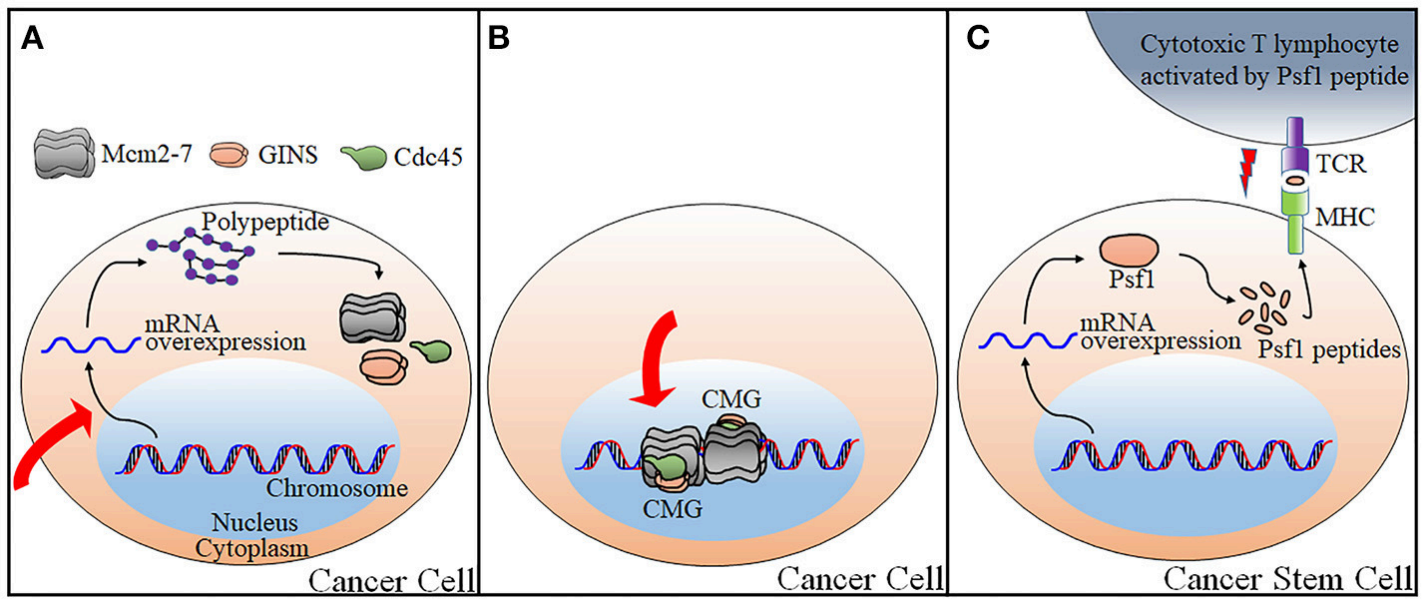
Current strategies applicable for targeting the CMG for anti-cancer therapy. Three possible strategies to control CMG are as illustrated. **(A)** Control of expression levels of the CMG subunits either by transcriptional or post-transcriptional regulations. **(B)** Control of CMG helicase by targeting the catalytic center of the CMG helicase or by disrupting the proein-protein interactions required for the CMG complex formation and activation. **(C)** CTL-mediated growth inhibition of cancer stem cells that overexpresses Psf1 by vaccination with antigenic Psf1 peptides. The red arrows in panels **A** and **B** indicate a process which is blocked by a small molecule. The red “lightening” symbol in panel **C** indicates death signal from CTL. TCR, T-cell receptor; MHC, Major histocompatibility complex.

Knockdown of the CMG subunits could be achieved by identification of small molecules exerting its effect on transcription or post-transcriptional regulation (Figure 2A). Changes of target genes at transcriptional levels could occur through direct association of such molecules with transcriptions factors, through induction of DNA damages, or through the alteration of signaling pathways governing downstream gene transcription as described below.

It was shown that genistein, a natural, nontoxic dietary isoflavone and trichostatin A (TSA), a classical histone deacetylase (HDAC) inhibitor downregulated Mcm2 expression in prostate cancer (Majid et al., 2010). These molecules also decreased expression levels of protein factors required for loading and activation of Mcm2-7, which include Cdt1, Cdc7, and Cdk2. The same study showed that levels of microRNA (miR)-1296 expression was reduced in carcinoma samples and miR-1296 targeted Mcm2 mRNA probably by binding to the 3′ untranslated region (3′ UTR) of Mcm2. Expression of miR-1296 was enhanced by treatment of PC3 cells with genistein. Treatment with genistein, and TSA, or transfection of miR-1296 to PC3 cells all reduced the percentage of S-phase cells.

Other independent studies confirmed the inhibitory effect of TSA on Mcm2 expression in HCT116 colon cancer cells (Liu et al., 2013). Real-time PCR (RT-PCR) arrays of HCT116 cells revealed that 34 cell cycle-related genes were significantly changed with regard to mRNA expression level in the presence of TSA. Among them, the expression level of Mcm2 was considerably reduced. Treatment of TSA induced growth inhibition and apoptosis in human HCT116 cells and the same phenotype was induced by transfection of Mcm2-specific siRNA. It was reported that JNK signaling is activated by TSA (Han et al., 2010). Treatment of a JNK inhibitor, SP600125 in HCT116 cells restored TSA-induced decrease of Mcm2, suggesting that the JNK signaling pathway is responsible for downregulation of Mcm2 (Liu et al., 2013).

Widdrol, an aromatic compound derived from *Juniperus chinensis* induced G1 phase arrest and inhibited growth of some cancers including A549 lung carcinoma and HT29 colon adenocarcinoma (Hong et al., 2009; Kwon et al., 2010). IC_50_ of widdrol was lowest in HT29 cells among cell lines tested (Kwon et al., 2010). In A549 lung carcinoma cells, levels of p53 and p21, a Cdk inhibitor increased, while those of Cdk2, cyclin E, retinoblastoma protein (RB), PCNA, and all Mcm subunits decreased (Hong et al., 2009). In HT29 colon adenocarcinoma cells, ataxia-telangiectasia mutated kinase (ATM) and p21 were upregulated and Cdc25A, Cdk2, cyclin E, E2F7, PCNA and the Mcm2-7 were downregulated (Kwon et al., 2010; Yun et al., 2012). Chk2 and p53 were activated by DNA damages that were probably directly induced by widdrol treatment in HT29, which leads to upregulation of p21 and reduced phosphorylation of RB. This result suggests that upregulated p21 exerts an inhibitory effect on the Cdk2/cyclin E complex which is required for inactivating phosphorylation of RB. Activated RB prevents E2F-mediated transcription, thereby decreasing expression of downstream genes including Mcms. Decrease of Mcm4 was detected in early time points after treatment of widdrol in HT29.

Statin family drugs include inhibitors of 3-hydroxy-3-methylglutaryl coenzyme A (HMG-CoA) reductase used for lowering plasma cholesterol levels (Zhang X. et al., 2015). However, statins have been considered as candidates for anti-cancer drug because of its ability to arrest cell cycle, to suppress tumor growth, and to induce apoptosis. It was shown that atorvastatin inhibited expressions of Mcm6 and Mcm7 in rat aortic vascular smooth muscle cells by inhibiting E2F promoter activity (Bruemmer et al., 2003). Treatment of lovastatin inhibited growth of NSCLC cells, arrested cell cycle at G1/S phase, and induced apoptosis (Zhang X. et al., 2015). Lovastatin activated the JNK signaling pathway involved in the downregulation of Mcm2 as observed with TSA treatment described above. Addition of the JNK inhibitor SP600125 to A549 and GLC-82 cells restored lovastatin-induced downregulation of Mcm2.

Although Metformin (N′,N′-dimethylbiguanide) was first developed to treat type2 diabetes, recent studies demonstrated that it has anticancer activity (Kim et al., 2017). Metformin was found to inactivate mTOR, thereby leading to activation of p53 and cell cycle arrest. Treatment of metformin to colorectal cancer cell lines downregulated expression of genes involved in cell cycle regulation and DNA replication including Mcms and PCNA in colorectal cancer cells resistant to 5-fluorouracil (5-FU, explained below).

BET is the abbreviation of bromodomain and extra-terminal family of proteins (Nicodeme et al., 2010). Proteins, which belong to this family, recognize acetylated histones for transcription of downstream target genes. I-BET (or BETi) is a synthetic compound developed to mimic acetylated histones in order to disrupt chromatin complexes responsible for expression of genes involved in inflammation. Several BETis were developed and some of them were shown to inhibit proliferation of cancer cells. In leukemic cells, repression of the c-MYC oncogene appears to be responsible for the antiproliferative activity of BETis (Ott et al., 2012). In one study, it was shown that treatment of BETis in anaplastic thyroid carcinoma (ATC) cell lines induced S-phase arrest and cell death (Mio et al., 2016). Significant increases in Mcm5 levels were detected both in human and mouse ATC cells compared to normal thyroids. Transcriptome analysis revealed that Mcm5 belonged to the top 20 downregulated genes in ATC cells treated with BETis.

Breviscapine (BVP) is a natural flavonoid, extracted from the Chinese herb *Erigerin breviscapus*. It has been demonstrated that BVP suppressed growth of human prostate cancer cell lines and tumors in mouse xenograft experiments (Guan et al., 2017). BVP reduced Mcm7 expression and authors claimed that this was associated with DNA damage in prostate cancer cells and tumor tissues, although detailed mechanism still remains unclear. BVP also induced apoptosis of cancer cells through the caspase-3 pathway.

The second theoretic mechanism involved in targeting Mcms for anti-cancer therapy described above could be used for combinatorial therapy, that is, knockdown of a constituent of the CMG in the presence of a drug that gives replicative stress to cancer cells. Gemcitabine and 5-FU have been widely used to treat several cancers (Longley et al., 2003; Brown et al., 2014). Gemcitabine is a nucleoside analog in which two hydrogen atoms at the C-2 position of deoxycytidine are replaced by fluorine atoms and 5-FU is an uracil analog which has a fluorine atom at the C-5 position in place of hydrogen. Both drugs induce DNA replication fork stalling by terminating DNA strand elongation by polymerases or by negatively affecting deoxyribonucleotide (dNTP) pool required for DNA synthesis (Longley et al., 2003; de Sousa Cavalvante and Monteiro, 2014). In one study, a combination therapy using siRNA against Mcms and the chemotherapeutic drug was attempted to increase treatment efficacy in pancreatic ductal adenocarcinoma (PDAC) (Bryant et al., 2015). Reduction of Mcm4 or Mcm7 sensitized PDAC cells to gemcitabine and 5-FU by preventing the formation of Mcm complex reservoir required to activate backup origins.

Screening and modification of small molecules modulating CMG expression and development of delivery materials of small molecules and siRNAs against components of the CMG including nanoparticles and conjugate delivery systems will improve targeting the CMG for cancer therapy (Kanasty et al., 2013).

### Strategy II. inhibition of enzymatic activity of the CMG complex

The second strategy for targeting the CMG is to directly inhibit enzymatic activity of the CMG complex (Figure 2B). There are a few small molecules identified that are able to inhibit helicase activity of the Mcm complexes including the Mcm2-7 and the Mcm4/6/7.

Heliquinomycin was originally discovered from *Streptomyces* species using helicase assays carried out with fractionated nuclear extracts from HeLa cells for the purpose of isolating DNA helicase inhibitors (Chino et al., 1996). Heliquinomycin was demonstrated to have antibiotic effects on microorganisms and inhibited DNA replication and RNA synthesis in cultured cancer cell lines (Chino et al., 1996, 1998). Ishimi and his colleagues performed DNA unwinding assays using several candidate helicases in the presence of heliquinomycin to identify target helicases of heliquinomycin and found that inhibitory effect was most dramatic with human Mcm4/6/7 compared to other helicases including SV40 large T antigen and Werner helicase (Ishimi et al., 2009). The IC_50_ value of heliquinomycin in the inhibition of Mcm4/6/7 was 2.4 μM and it was similar to that of heliquinomycin in inhibiting cellular DNA replication. The inhibitory effect of heliquinomycin on ATPase activity of the Mcm4/6/7 was observed only in the presence of ssDNA. Heliquinomycin also effectively suppressed the growth of cancer cells including lung adenocarcinoma, lung large cell carcinoma, and bladder cancer cells (Toyokawa et al., 2011).

Fluoroquinolones are a group of antibiotics targeting bacterial gyrase and topoisomerase IV enzymes, and it was reported that they possess inhibitory effect on the helicase activity of SV40 large T antigen (Ali et al., 2007). Among fluoroquinolones, ciprofloxacin displayed selective inhibition toward yeast Mcm2-7 (Simon et al., 2013). While ofloxacin inhibited both the Mcm2-7 and the Mcm4/6/7 with similar IC_50_ values, 4.17 and 5.29 mM, respectively, ciprofloxacin inhibited the Mcm2-7 helicase activity more efficiently (Mcm2-7, IC_50_ = 0.63 mM; Mcm4/6/7, IC_50_ = 1.89 mM). The IC_50_ of ciprofloxacin to SV40 large T antigen was higher (4 mM) that those with the Mcm helicases. Thus, ciprofloxacin appears selective toward the Mcm2-7 from human and yeast cells and its selectivity was further verified by examining its cytotoxicity. This holds promises in designing drugs that target a specific helicase only. In this case, the CMG complex could be such a specific target.

It has been pointed out that the CMG complexes are difficult to purify in sufficient amounts for extensive high-throughput screening application and only a few groups succeeded to purify the CMG (Moyer et al., 2006; Kang et al., 2012; Georgescu et al., 2014; Simon and Schwacha, 2014; Abid Ali et al., 2016; Zhou et al., 2017). The yield of human CMG was ~40 pmol (0.03 mg) from 2-liter Sf9 insect cells, and 0.3 mg of yeast CMG was purified from 12-liter culture of overexpressing haploid yeast strain (Kang et al., 2013; Georgescu et al., 2014). Recently, significant large amounts of yeast CMG have been successfully purified from a diploid strain (Zhou et al., 2017). Therefore, initial screenings of small molecules that inhibit helicase or ATPase activities could be performed with yeast CMG, followed by subsequent validation of the hit molecules with human CMG. On the other hand, it would be worthwhile to construct a yeast strain co-expressing 11 subunits of human CMG. This would allow to increase the purification scale to obtain CMG in large amounts enough for high-throughput screenings.

### Strategy III. cancer vaccine

The third strategy is not intended to regulate either expression levels of the CMG constituent or to inhibit enzymatic activity of the CMG complex. Rather, it targets a CMG constituent with somewhat different ways and it is irrelevant to the regulation of the CMG action on DNA replication. Cancer immunotherapy triggers the immune system by recognizing molecular entities expressed specifically on the surface of cancer cells to eliminate these cells (Yoshida et al., 2017). Cytotoxic T lymphocytes (CTLs) attack cancer cells by recognizing cancer-specific antigenic peptides presented with human leukocyte antigen (HLA) on the cell surface. Recently, Yoshida and his colleagues has identified a Psf1-derived peptide presented by HLA through bioinformatics approach and mass spectrometric analyses. The HLA-Psf1 peptide complex was purified from breast cancer cell lines by overexpressing both HLA and Psf1 and this attempt resulted in the identification of Psf1_79−87_ peptide. Splenocytes obtained from HLA expressing transgenic mice vaccinated with Psf1_79−87_ peptide secreted interferon-γ when incubated with T2 cells pre-incubated with Psf1_79−87._ In addition, CTLs generated by peptide stimulation of peripheral blood mononuclear cells from human blood were purified, followed by co-culture with monocyte-derived dendritic cells pulsed with Psf1_79−87_. The peptide-specific CTLs killed Psf1_79−87_-pulsed T2 cells more effectively than non-pulsed cells (2.4 fold), indicating that vaccination is effective in inducing immune response (Figure 2C). This result is promising because the same group also reported a close association of Psf1 overexpression with cancer stem cells (Nagahama et al., 2010b). Previously, the same strategy had also been applied to Cdc45 (Tomita et al., 2011). Highly immunogenic Cdc45-derived peptides induced CTLs to be reactive to lung cancer cells. Further studies are required to evaluate this technique in clinical trial for targeting cancer stem cells.

## Prospects for new strategies targeting the CMG

Development of novel strategies targeting the CMG will shed light on the field of cancer therapy. Currently, most strategies are based on the regulation of protein expression and control of enzymatic activities by small molecules. However, peptides and antibodies, could be used as an alternative to small molecule-based therapeutics in downregulating CMG activity.

Depletion strategy has been tested not only with Mcms, but also with other subunits of CMG. It has been proposed that 60% of total genes in humans are regulated by miRNA, and it was recently discovered that a miRNA governs Sld5 expression and Sld5 was robustly expressed in human bladder cancer due to the reduced level of a candidate miRNA that interacts with the 3′-UTR of the SLD5 gene (Yamane et al., 2016). The candidate miRNA turned out to be miRNA-370 and was downregulated in the bladder cancer cells. Overexpression of IL-6 (Interleukin 6) was also observed and enhanced expression of DNA-methyltransferase1 (DNMT1), leading to suppression of miR370, which in turn resulted in overexpression of Sld5. Thus, downregulation of IL-6 by siRNA could suppress Sld5 expression in T24 bladder cancer cell line and injection of miR-370 could inhibit tumor growth in the mouse xenograft experiment. IL-6 is a valuable target for treatment of dysimmune diseases and cancers (Rossi D. et al., 2015; Rossi J. F. et al., 2015). Many antagonistic monoclonal antibodies against IL-6 and the IL-6 receptor are under clinical trials and several of them were shown to be effective in certain diseases including rheumatoid arthritis. Therefore, bladder cancer could be tested for anti-IL-6 therapies to find whether tumor growth could be suppressed by preventing the autocrine loop of cancer cells which leads to Sld5 downregulation. Alternatively, sgp130, a natural inhibitor for IL-6 signaling could also be used for the inhibition of cancer growth (Hong et al., 2016).

The inhibition of the CMG helicase activity could take place in a variety of ways. For example, ciprofloxacin is likely to negatively interact with the ATPase active sites of the Mcm2-7 helicase to inhibit its helicase activity, although how it does is not clear at present (Simon et al., 2013). Small molecules could inhibit the CMG helicase activity via their competitive binding with the ATP binding pocket, interfering with the CMG-DNA interaction or via disruption of subunit interactions. PCNA is similar to the CMG in terms of its role in cell proliferation and acts as a marker for cancer. Therefore, studies on the PCNA inhibitor could give insight into development of the CMG inhibitors. Several compounds and peptides have been reported to inhibit the PCNA functions (Wang, 2014). PCNA forms a ring-shaped homotrimeric complex that encircles DNA double-helix and functions as a processivity factor for DNA polymerases, and it coordinates DNA synthesis by direct interactions with other replication proteins including ligases and topoisomerases. PCNA also has diverse roles in many cellular pathways including DNA damage repair, DNA damage avoidance pathway, cell cycle regulation, chromatin assembly, and transcription. Cellular reactions mediated by the PCNA usually occur through recruitment of the PCNA binding proteins on each subunit of the PCNA trimer. The interdomain-connecting loop (IDCL) is the major constituent participating in the PCNA-protein interaction by binding to the PCNA-interaction protein (PIP) box in the binding proteins. Several small molecules and peptides were identified that could inhibit the IDCL-PIP box interaction. For example, the PIP-box containing peptide derived from p21 inhibited DNA replication and cell growth (Pan et al., 1995; Warbrick et al., 1995; Chen et al., 1996). In addition, compounds that inhibit formation of the PCNA trimer by docking at the monomer interface of the PCNA and a peptide that prevents phosphorylation of the PCNA Y211 inhibited cancer growth (Zhao et al., 2011; Tan et al., 2012).

**Figure 3:**
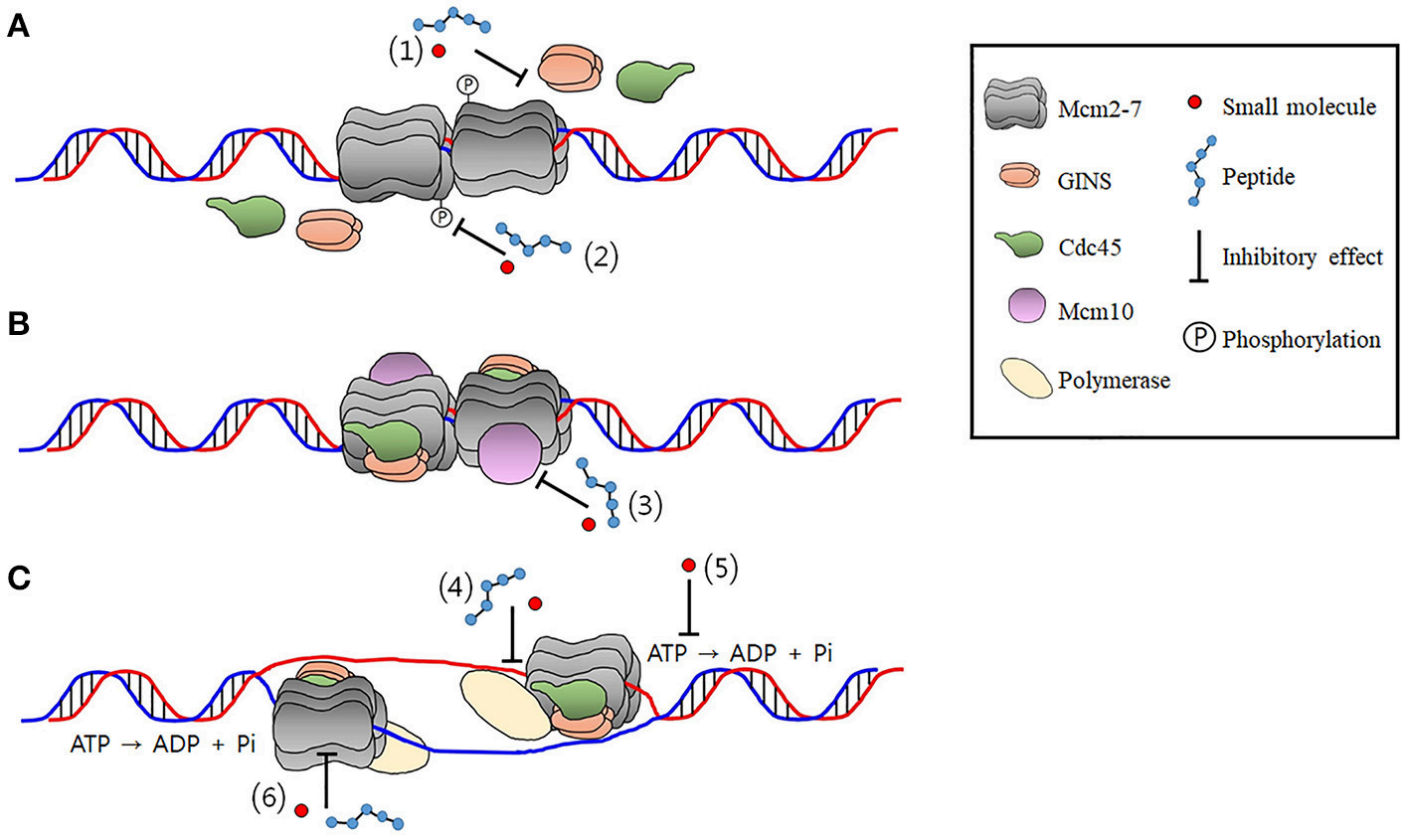
The potential CMG-targeting sites of small molecules or peptides. The findings that multiple essential steps are required to form a functional CMG complex make the CMG a target well suited to develop drugs with a number of different inhibition mechanisms. These include inhibition of **(A)** formation, **(B)** activation, and **(C)** prevention of the catalytic activity of the CMG complex. The number in paranthesis denotes a site of inhibition. (1) Inhibition of the CMG formation by preventing interactions among the Mcm2-7, Cdc45, and the GINS. Inhibition of phosphrylation of Sld2/RecQL4 and Dpb11/TopBP1 by CDK and protein-protein interactions that occur in the precess of the CMG formation is not shown for simplicity. (2) Inhibition of the Mcm2-7 phosphorylation by DDK required for the CMG formation. (3) Inhibition of Mcm10 required for the activation of the CMG. (4) Uncoupling of the CMG-polymerase interactions during initiation and elongation stage of DNA replication. (5) Inhibition of enzymatic activity of the CMG required for duplex unwinding by targetting catalytic center of Mcms or residues required for translocation on DNA. (6) Disruption of intermoleculr interactions in the CMG.

The CMG is an 11-subunit complex and complex formation is essential for its helicase activation and cell proliferation. Furthermore, the CMG formation and activation is a multistep process that requires a variety of protein-protein interactions. Therefore, designing of small molecules and peptide inhibitors targeting inter-subunit interfaces within the CMG complex and protein-protein interactions between the CMG and other replication factors has immense importance in development novel anti-cancer drugs. It has been shown that the N-terminal peptides of the RB protein suppressed CMG helicase in *Xenopus* egg extract experiment, although it needs to be verified with the purified CMG complex (Borysov et al., 2015). Other Mcm-interacting proteins that have potentials to block replication or checkpoint activation, reviewed elsewhere, could be candidates for designing peptide drugs inhibiting the CMG activity (Simon and Schwacha, 2014). For example, prohibitin protein, which suppressed E2F-mediated transcription to inhibit cell proliferation, was shown to physically interact with Mcms and inhibit DNA replication *in vivo* (Rizwani et al., 2009). Posttranslational modifications of proteins also could be targeted as in the case of PCNA, because phosphorylation of Sld3/Treslin and Sld2/RecQL4 by CDK and phosphorylation of Mcms by DDK are essential steps for the CMG formation and stimulation (Tanaka and Araki, 2013). In addition, phosphorylation of Mcm7 increases complex formation with other Mcms and enhances chromatin binding, resulting in cell proliferation. Potentiation of Mcm7 phosphorylation has been observed in several cancers (Huang et al., 2013; Fei et al., 2017). Sites that could be targeted by small molecules and peptides during the formation and activation of CMG as well as replication initiation are illustrated in Figure 3.

Helicases are emerging targets that have great potential for the treatment of serious human proliferative diseases such as cancers. Identification of novel-type drugs targeting CMG, the core of DNA replication and cell proliferation, is of immense importance in developing new therapeutics against cancers. For this purpose, we may need more understanding of diverse features of the CMG complex that includes structural aspects, complex assembly and activation, mechanism of action, involvement with checkpoint, and intracellular and extracellular factors affecting transcriptional regulation of the CMG. The more information in these regards we obtain, the more possibility we will have for better drug design. Development of specific assays for assessing each step of CMG assembly, testing protein-protein interactions, and the success of high-yield purification of the CMG combined with high-throughput drug screenings will provide innovative means to identify small molecules targeting CMG assembly and helicase activity, ultimately contributing to development of novel and effective cure for human cancers.

## Author contributions

Both Y-SS and Y-HK conceived and outlined the manuscript. Y-HK wrote the main text and produced the tables and figures. Y-SS critically reviewed and revised the manuscript.

### Conflict of interest statement

The authors declare that the research was conducted in the absence of any commercial or financial relationships that could be construed as a potential conflict of interest.
